# Pain chronification and the important role of non-disease-specific symptoms in patients with systemic sclerosis

**DOI:** 10.1186/s13075-021-02421-1

**Published:** 2021-01-19

**Authors:** Caroline Evers, Suzana Jordan, Britta Maurer, Mike Oliver Becker, Carina Mihai, Rucsandra Dobrota, Petra Hoederath, Oliver Distler

**Affiliations:** 1 Department of Rheumatology, University Hospital Zurich, University of Zurich, Gloriastrasse 25, 8091 Zurich, Switzerland; 2 Centre of Neurosurgery Hirslanden Ostschweiz, Paintherapy Stephanshorn, Brauerstrasse 95a, 9016 St. Gallen, Switzerland

**Keywords:** Systemic sclerosis, Pain, Chronification, Non-disease-specific symptoms

## Abstract

**Background:**

Pain is a frequent, yet inadequately explored challenge in patients with systemic sclerosis (SSc). This study aimed to conduct an extensive pain assessment, examining pain chronification and its association with disease manifestations.

**Methods:**

Consecutive SSc patients attending their annual assessment were included. SSc-specific features were addressed as defined by the European Scleroderma Trials and Research (EUSTAR) guidelines. Pain analysis included intensity, localization, treatment, chronification grade according to the Mainz Pain Staging System (MPSS), general well-being using the Marburg questionnaire on habitual health findings (MFHW) and symptoms of anxiety and depression using the Hospital Anxiety and Depression Scale (HADS).

**Results:**

One hundred forty-seven SSc patients completed a pain questionnaire, and 118/147 patients reporting pain were included in the analysis. Median pain intensity was 4/10 on a numeric rating scale (NRS). The most frequent major pain localizations were hand and lower back. Low back pain as the main pain manifestation was significantly more frequent in patients with very early SSc (*p* = 0.01); those patients also showed worse HADS and MFHW scores. Regarding pain chronification, 34.8% were in stage I according to the MPSS, 45.2% in stage II and 20.0% in stage III. There was no significant correlation between chronification grade and disease severity, but advanced chronification was significantly more frequent in patients with low back pain (*p =* 0.024). It was also significantly associated with pathological HADS scores (*p* < 0.0001) and linked with decreased well-being and higher use of analgesics.

**Conclusions:**

Our study implies that also non-disease-specific symptoms such as low back pain need to be considered in SSc patients, especially in early disease. Since low back pain seems to be associated with higher grades of pain chronification and psychological problems, our study underlines the importance of preventing pain chronification in order to enhance the quality of life.

## Background

Systemic sclerosis (SSc) is a rare autoimmune connective tissue disease. The pathophysiology is not yet fully understood but includes vascular and fibrotic changes. Endothelial, fibroblast and immune system dysregulation results in vasculopathy, immune cell activation, collagen accumulation and the presence of autoantibodies. Clinical manifestations are heterogeneous; frequent manifestations are Raynaud’s phenomenon and digital ulcers, skin fibrosis and joint involvement. Various symptoms may result from organ involvement of the gastrointestinal tract, lungs, heart or kidneys [1, 2].

Pain is a frequent symptom in SSc patients and can have various causes. It may for example derive from vasospasms, digital ulcerations, synovitis, joint contractures or gastrointestinal dysmotility. Despite the fact that it is an important concern for these patients, there is only very limited data available about pain in SSc [3–6]. Pain has a major impact on the patient’s activities by impairing physical function [7, 8]. Previous studies have found that patients with SSc show significantly impaired health-related quality of life compared to the general population [9–11]. Several studies found that impaired quality of life is essentially associated with pain in SSc patients, emphasizing the importance of adequate pain therapy in the patient’s treatment [12–14]. Furthermore, symptoms of anxiety and depression are frequent in SSc patients and are associated with decreased health-related quality of life [15]. A review found that between 36 and 65% of all SSc patients show clinically significant symptoms of depression [16]. Since pain is significantly associated with depressive symptoms in SSc [7, 14], screening for and treatment of pain is of great importance for these patients.

Despite SSc being a chronic and non-curable disease, chronification of pain has not been addressed in SSc. Chronic pain is an important issue in the general population with a prevalence between 10 and 44% [17]. A large survey about chronic pain in Europe showed an overall point prevalence of 19% and a prevalence of 16% for Switzerland, with back pain existing in almost half of the patients [18]. The wide ranges of prevalence are mainly caused by the lack of a standardized definition. Chronic pain is often defined as pain that persists for more than 3 [19] to 6 months [20]. Sometimes, chronic pain is defined as pain that persists for longer than the expected duration of healing [19]. More recent findings imply that pain chronification should be considered as a multidimensional process with increasing physical, psychological and social problems leading to significant limitations in daily activities [20]. Chronic pain is associated with depressive symptoms [21, 22] and a meaningfully decreased health-related quality of life [23–25]. A more recent literature-based study from 2013 highlights the high prevalence, individual and societal impact of chronic pain as well as problems with inadequate treatment and the need for pain treatment education and guidelines [26].

To assess pain chronification, the Mainz Pain Staging System (MPSS) is frequently used, showing good validity in patients with different pain diagnoses [27–29]. This model considers the multidimensional aspect of chronic pain, using information on chronological and spatial qualities of pain as well as information on medication and the patient’s history. It differentiates between three stages of pain chronification. Higher stages correspond to increased pain chronification, typically associated with permanent, multilocular pain, as well as high usage of medication and resources of the health system.

In this study, we conducted a detailed pain analysis in order to learn more about the associations between pain characteristics, chronification and disease manifestations in patients with SSc.

## Methods

### Patients

Consecutive patients with SSc attending the annual assessment programme at the Department of Rheumatology of the University Hospital Zurich were recruited for this study. All patients were part of the local European Scleroderma Trials and Research (EUSTAR) registry [30].

Patients who reported no pain at all, patients with the annual clinical assessment more than ± 3 months apart from the pain assessment and patients with rheumatoid arthritis overlap, potentially influencing pain symptoms particularly in the hands, were excluded from the analysis.

We used fulfilment of the different classification criteria as a measure of disease severity and assigned the patients to three groups: patients who fulfilled both the 1980 American College of Rheumatology (ACR) 1980 criteria [31] for SSc and the ACR/EULAR 2013 classification criteria formed the group “established” and patients fulfilling only the ACR/EULAR 2013 classification criteria [32] were ascribed to the group “mild”. Patients who fulfilled neither of these classification criteria but were clinically diagnosed with SSc by a clinical expert with long-term experience in SSc (OD and BM) based on the presence Raynaud’s phenomenon and at least one of the following features including puffy fingers, positive antinuclear antibodies or pathological nailfold capillaroscopy formed the group “very early”, representing a very early stage of the disease [33]. Further, we were interested to analyse how localization of pain affects the SSc patients; therefore, we compared patients with hand and back pain as major pain, since those were the most frequent localizations of major pain.

All patients included in this study had signed informed consent. The study had been approved by the Cantonal Ethics Committee (BASEC-Nr 2017-02115).

### Measures

#### Disease characteristics

Information on disease-related symptoms such as skin, joint and organ involvement was retrieved from the EUSTAR database and compared amongst the three groups “established”, “mild” and “very early” for patient characteristics. Clinical manifestations and organ involvement are defined according to EUSTAR standards [34]. Disease duration was measured according to the first occurrence of non-Raynaud’s symptoms. Clinical signs of arthritis were defined as at least two tender and swollen joints. Lung fibrosis had to be confirmed by high-resolution computed tomography (HRCT). Scleroderma pattern in nailfold capillaroscopy was defined as described [35]. To assess skin thickening, the Modified Rodnan Skin Score (mRSS) was used (a scoring system of 17 body regions, with maximal score per region of 3 points and maximal mRSS of 51 points) [36].

#### Hand function

Hand function was assessed with the Cochin Hand Function Scale (CHFS), which was found to be a valid and reliable tool in patients with SSc [37, 38]. It consists of 18 questions about hand function concerning dressing, hygiene, hand ability in the kitchen and at the office and other daily activities (with 5 levels of difficulty, from none to impossible).

#### Pain characteristics

In this study, the German pain questionnaire was used, which was confirmed to be a valid and reliable tool for pain assessment [27]. The questionnaire was available in German, English, French and Italian; the patients could choose the language they were most familiar with. The questionnaire contains information on pain localization, duration, intensity, character and therapies, as well as questions concerning general well-being and social situation. Pain intensity is assessed with an 11-point numeric rating scale (NRS) from 0 to 10, with 0 being no pain and 10 being the worst pain imaginable. Concerning pain localization, patients were asked to distinguish between their major pain, which has the most severe impact on their life and well-being, and other pain localizations. Regarding pain medication, analgesics were differentiated according to the World Health Organization (WHO) pain ladder [39]. WHO stage I consists of non-opioids, stage II of mild opioids (e.g. tramadol, codeine) and stage III of strong opioids (e.g. oxycodone, morphine, fentanyl). Further, adjuvant medications such as antidepressants, anticonvulsants and topical medication were recorded. In the last part of the questionnaire, the pain chronification grade was assessed using the MPSS, which showed good construct validity in previous studies [27–29]. To differentiate between the three stages of chronification, this model considers four axes. Chronological aspects are assessed with questions about pain duration, frequency and change of intensity. Spatial aspects are considered differentiating whether pain is localized in one, two or more body sites. Regarding medication, the use of analgesics and potential withdrawal therapies are assessed. Last, the patient’s personal history with changes of doctors, pain-related hospitalizations, surgery and rehabilitation programmes is recorded. From the four axes’ sum scores, the total score and by this, the chronification stage was calculated.

#### Psychological aspects

Psychological factors were assessed using the Hospital Anxiety and Depression Scale (HADS), which was shown to be a good screening instrument for anxiety and depressive symptoms in patients with non-psychiatric illnesses [40]. This scale was designed especially for physically ill patients and does not contain any somatic symptoms, since they could be caused by the underlying disease and lead to overestimation [41]. It consists of 7 questions each for anxiety and depression, resulting both in a sum score from 0 to 21. In our study, we considered scores of 11 and more as pathologic and scores between 8 and 10 as borderline.

The Marburg questionnaire on habitual health findings (MFHW) [42] was used to assess the patient’s general well-being, resulting in a score from zero to 35 with lower scores corresponding to bad well-being. Scores of 10 and below were considered pathological.

### Statistical analysis

For statistical analysis, IBM SPSS Statistics, version 2.5, was used. For nominal data, frequencies were calculated, and chi-square test or Fisher’s exact test was used for comparison. For ordinal or continuous data, means with standard deviation (SD) were calculated in case of normative distribution; otherwise, medians with first and third quartiles were calculated. For comparison of ordinal or continuous variables, the Mann-Whitney *U* test was used for two groups and the Jonckheere-Terpstra test was used for more than two groups. A *p* value smaller than 0.05 was considered significant.

## Results

### Patient characteristics

Altogether 147 SSc patients completed the questionnaires. Nineteen patients were excluded due to lack of pain, eight because the clinical assessment was more than ± 3 months apart from the pain assessment, one due to overlap with rheumatoid arthritis and one because the patient did not have SSc. Thus, 118/147 (80.1%) patients reporting pain could be included in this study. Their clinical characteristics are summarized in Table 1. The majority of them were females (104/118, 88.1%). The mean age was 57 (± 13.7 SD) years. Sixty-five (55.1%) patients fulfilled the 1980 ACR criteria for SSc and were allocated to the group “established”. Twenty-nine (24.6%) fulfilled only the 2013 classification criteria and were assigned to the group “mild”. The remaining 24 (20.3%) patients, who did not yet fulfil any of these criteria, but had an expert diagnosis of SSc, formed the group “very early”. The group “established” contained 31/65 (47.7%) patients with diffuse SSc (dSSc) according to the classification of LeRoy et al. [43], whilst there were only patients with limited cutaneous SSc (lcSSc) in the group “mild”. As expected, the group “established” showed more symptoms typical for SSc with more severe skin involvement, worse hand function and lung organ involvement. Disease manifestations associated with pain such as active and previous digital ulcers (18.5% vs. 3.4%, *p* = 0.006, and 40.0% vs. 17.2%, *p* < 0.001, respectively), joint contractures (51.6% vs. 18.2%; *p* = 0.002) and subcutaneous calcinosis of the hand (18.0% vs. 3.6%; *p* = 0.008) were significantly more frequent in the “established” group than in the “mild” group. In the “very early” group, digital ulcers and subcutaneous calcinosis were not present.

**Table 1:** Patient characteristics

Subset of disease manifestation	Overall, *n* = 118	Established, *n* = 65 (55.1%)	Mild, *n* = 29 (24.6%)	Very early, *n* = 24 (20.3%)
Age (years)	57.1 ± 13.7	56.1 ± 13.6	64.1 ± 9.1	51.3 ± 15.4
Female	104/118 (88.1%)	53/65 (81.5%)	28/29 (96.6%)	23/24 (95.8%)
Disease duration (months)	–	82 _(42, 246)_, *n* = *64*	80 _(33, 142)_, *n* = *21*	Not applicable
Subset according to LeRoy				
lcSSc	–	34/65 (52.3%)	21/21 (100%)	Not applicable
dSSc	–	31/65 (47.7%)	0/21 (0.0%)	Not applicable
Extent of skin involvement (current)				
No skin involvement	43/118 (36.4%)	2/65 (3.1%)	19/29 (65.5%)	22/24 (91.7%)
Only sclerodactyly	12/118 (10.2%)	4/65 (6.2%)	7/29 (24.1%)	1/24 (4.2%)
Limited cutaneous involvement	44/118 (37.3%)	40/65 (61.5%)	3/29 (10.3%)	1/24 (4.2%)^1^
Diffuse cutaneous involvement	19/118 (16.1%)	19/65 (29.2%)	0/29 (0.0%)	0/24 (0.0%)
mRSS	2.5 _(0, 9)_, *n = 118*	8.0 _(4, 15)_, *n = 65*	0.0 _(0, 2)_, *n = 29*	0.0 _(0, 0)_, *n = 24*
Cochin Hand Function Scale	2.0 _(0, 12)_, *n = 65*	4.5 _(0, 13)_, *n = 48*	0.0 _(0, 4)_, *n = 21*	0.0 _(0, 4)_, *n = 16*
Disease characteristics				
Raynaud’s phenomenon present	115/118 (97.5%)	63/65 (96.9%)	28/29 (96.6%)	24/24 (100%)
Puffy fingers (current)	47/101 (46.5%)	29/48 (60.4%)	17/29 (58.6%)	1/24 (4.2%)
Digital ulcers (current)	13/118 (11.0%)	12/65 (18.5%)	1/29 (3.4%)	0/24 (0.0%)
Digital ulcers (previously)	31/118 (26.3%)	26/65 (40.0%)	5/29 (17.2%)	0/24 (0.0%)
Joint synovitis	24/118 (20.3%)	11/65 (16.9%)	11/29 (37.9%)	2/24 (8.3%)
Clinical signs of arthritis	14/102 (13.7%)	4/51 (7.8%)	8/28 (28.6%)	2/23 (8.7%)
Joint contractures	37/90 (41.1%)	33/64 (51.6%)	4/22 (18.2%)	Not available
Tendon friction rubs	5/111 (4.3%)	4/64 (6.3%)	1/28 (3.6%)	0/24 (0.0%)
Subcutaneous calcinosis (hands)	10/101 (9.9%)	9/50 (18.0%)	1/28 (3.6%)	0/23 (0.0%)
Laboratory				
ANA	116/118 (98.3%)	64/65 (98.5%)	29/29 (100%)	23/24 (95.8%)
Anti-centromere	57/116 (49.1%)	19/63 (30.2%)	22/29 (75.9%)	16/24 (66.7%)
Anti-Scl-70	23/118 (19.5%)	22/65 (33.8%)	1/29 (3.4%)	0/24 (0.0%)
Anti-RNA polymerase III	12/117 (10.3%)	10/64 (15.6%)	0/29 (0.0%)	2/24 (8.3%)
Anti-U1nRNP	2/116 (1.7%)	2/64 (3.1%)	0/28 (0.0%)	0/24 (0.0%)
Anti-PMScl	8/111 (7.2%)	7/59 (11.9%)	0/28 (0.0%)	1/24 (4.2%)
CRP elevation	15/117 (12.8%)	10/64 (15.6%)	3/29 (10.3%)	2/24 (8.3%)
Organ involvement^2^				
Lung				
Dyspnoea present	53/116 (45.7%)	34/64 (53.1%)	13/29 (44.8%)	6/23 (26.1%)
Lung fibrosis on HRCT	39/115 (33.9%)	35/62 (56.5%)	4/29 (13.8%)	0/24 (0.0%)
Function				
DLCO (% predicted)	83.6 ± 18.9, *n = 116*	78.3 ± 20.0, *n = 63*	88.3 ± 16.6, *n = 29*	91.8 ± 14.3, *n = 24*
FVC (% predicted)	101.8 ± 18.6, *n = 117*	96.1 ± 18.9, *n = 64*	112.1 ± 16.7, *n = 29*	104.5 ± 13.9, *n = 24*
FEV-1 (% predicted)	95.6 ± 19.4, *n = 117*	90.1 ± 18.9, *n = 63*	103.3 ± 20.8, *n = 29*	100.8 ± 14.5, *n = 24*
TLC (% predicted)	100.9 ± 17.5, *n = 87*	98.2 ± 17.7, *n = 61*	106.9 ± 16.7, *n = 22*	Not available
GIT				
Oesophageal symptoms	60/118 (50.8%)	37/65 (56.9%)	15/29 (51.7%)	8/24 (33.3%)
Stomach symptoms	37/91 (40.7%)	26/65 (40.0%)	10/22 (45.5%)	Not available
Intestinal symptoms	38/91 (41.8%)	27/65 (41.5%)	10/22 (45.5%)	Not available
CV				
Palpitations	12/91 (13.2%)	9/65 (13.8%)	3/22 (13.6%)	Not available
Conduction blocks	11/96 (11.5%)	6/47 (12.8%)	4/26 (15.4%)	1/23 (4.3%)
LVEF (%)	63.1 ± 3.9, *n = 114*	63.1 ± 4.3, *n = 64*	63.6 ± 3.2, *n = 27*	62.5 ± 3.8, *n = 23*
Nailfold capillaroscopy				
SSc pattern	90/117 (76.9%)	59/64 (92.2%)	22/29 (75.9%)	9/24 (37.5%)
Immunosuppressive therapy^3^	26/118 (22.0%)	21/65 (32.3%)	4/29 (13.8%)	1/24 (4.2%)

Variables are presented as mean ± SD for normal distribution or as median with the 1st and 3rd quartiles _(Q1, Q3)_ for non-normal distribution. Frequencies are shown as *x*/*y*: *x* = number of patients with item present, *y* = numbers of patients with accessible data; missing values can be calculated *n* − *y*. Definition of clinical parameters and organ involvement according to EUSTAR standards [34]

*ANA* anti-nuclear antibody, *Anti-Scl-70* anti-topoisomerase I antibody, *CRP* C-reactive protein, *CV* cardiovascular, *dSSc* diffuse systemic sclerosis, *DLCO* diffusing capacity of the lung for carbon monoxide, *FEV-1* forced expiratory volume in one second, *FVC* forced vital capacity, *GIT* gastrointestinal tract, *HRCT* high-resolution computed tomography, *lcSSc* limited cutaneous systemic sclerosis, *LVEF* left ventricular ejection fraction, *mRSS* modified Rodnan Skin Score, *n* number, *RNA* ribonucleic acid, *SD* standard deviation, *SSc* systemic sclerosis, *TLC* total lung capacity, *TNF* tumour necrosis factor, *U1RNP* uridine-rich ribonucleic protein

^1^Skin fibrosis of the face

^2^In this cohort, none of the patients showed renal crisis

^3^Including prednisone, cyclophosphamide, methotrexate, azathioprine, mycophenolate, d-penicillamine, rituximab, imatinib mesylate and TNF-alpha inhibitors

### Pain analysis

#### Pain intensity does not increase with disease severity

Results for the pain analysis for the three groups “established”, “mild” and “very early” are displayed in Table 2. Overall, median pain intensity during the last 4 weeks was 4 on an NRS from 0 to 10. There was a numerically higher NRS in the established group, but this did not reach statistical significance (*p* = 0.199). The most frequent localizations patients reported as major pain were the hands and back, mainly referring to the lumbar spine. Further frequent pain localizations were the feet and additional joints of the lower extremity, showing no significant difference between the groups.

**Table 2:** General pain assessment

Subset of disease manifestation	Overall, *n* = 118	Established, *n* = 65 (55.1%)	Mild, *n* = 29 (24.6%)	Very early, *n* = 24 (20.3%)
Median pain intensity in the last 4 weeks (NRS)^1^	4.0 _(2, 5)_, *n = 115*	4.0 _(2, 6)_, *n = 64*	3.0 _(2, 5)_, *n = 27*	3.0 _(2, 5)_, *n = 24*
Duration since pain symptoms started				
Less than 1 year	19/112 (17.0%)	10/62 (16.1%)	8/27 (29.6%)	1/23 (4.3%)
1 to 5 years	55/112 (49.1%)	33/62 (53.2%)	11/27 (40.7%)	11/23 (47.8%)
More than 5 years	38/112 (33.9%)	19/62 (30.6%)	8/27 (29.6%)	11/23 (47.8%)
Most frequent localization for main pain				
Back pain	30/116 (25.9%)	14/65 (21.5%)	8/29 (27.6%)	8/22 (36.4%)
*Lumbar spine*	*19/116 (16.4%)*	*8/65 (12.3%)*	*5/29 (17.2%)*	*6/22 (27.3%)*
Hand	50/116 (43.1%)	32/65 (49.2%)	12/29 (41.4%)	6/22 (27.3%)
Most frequent localizations for overall pain				
Back	71/118 (60.2%)	35/65 (53.8%)	18/29 (62.1%)	18/24 (75.0%)
*Lumbar spine*	*46/118 (39.0%)*	*19/65 (29.2%)*	*12/29 (41.4%)*	*15/24 (62.5%)*
Hand	94/118 (79.7%)	52/65 (80.0%)	24/29 (82.8%)	18/24 (78.3%)
Joint of the lower extremity	67/118 (56.8%)	36/65 (55.4%)	14/29 (48.3%)	17/24 (70.8%)
Foot	40/118 (33.9%)	23/65 (35.4%)	10/29 (34.5%)	7/24 (29.2%)

Variables are presented as mean ± SD for normal distribution or as median with the 1st and 3rd quartiles _(Q1, Q3)_ for non-normal distribution. Frequencies are shown as *x*/*y*: *x* = number of patients with item present, *y* = numbers of patients with accessible data; missing values can be calculated *n* − *y*

*n* number, *NRS* numeric rating scale, *SD* standard deviation

^1^Referring to major pain

#### Hand pain is more frequent in advanced disease and associated with disease-specific symptoms

**Fig. 1:**
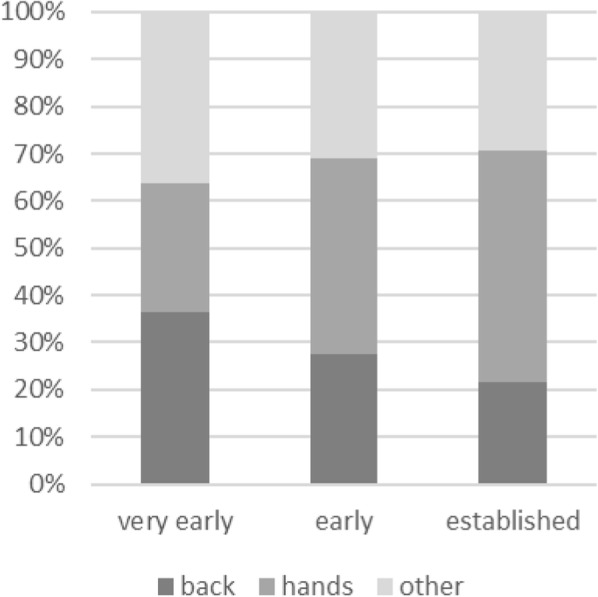
Frequencies for the main pain localizations compared between the groups “very early”, “mild” and “established”, showing a decrease of back pain and an increase of hand pain with progressing disease manifestation

As expected for SSc patients, the hands were one of the most frequent localizations of pain. Hand pain in general was reported by approximately 80% of the patients in all three groups. As shown in Table 2 and Fig. 1, there was a difference in the frequency of hand pain as major pain with 27.3% in the group “very early”, 41.4% in the group “mild” and 49.2% in the group “established”, although the difference was not found to be statistically significant (*p* = 0.150). Table 3 displays a direct comparison of patients with main pain in the hands vs. the lower back. Patients with hand main pain were more often classified “established” compared to patients with low back main pain. The median CHFS was 7.0; patients with hand pain as main pain had higher scores for CHFS (*p* = 0.001) than other patients; the same applied for patients who reported hand pain in general (*p* < 0.001). Patients reporting main pain localized in the hands had more frequently digital ulcers (*p* = 0.008), joint contractures (*p* = 0.003) and tendon friction rubs (*p* = 0.013) than other patients. Similarly, in patients with hand pain, the median pain intensity was higher when painful digital ischemia (*p* = 0.018; data not shown) or joint contractures (*p* = 0.019) were present.

**Table 3:** Hand pain compared to low back pain

Differences in main pain localization	Hand pain (*n* = 50)	Low back pain (*n* = 19)
Age (years)	55.4 ± 14.5	59.0 ± 13.1
Subset of disease manifestation		
Very early	6/50 (12.0%)	6/19 (31.6%)
Mild	12/50 (24.0%)	5/19 (26.3%)
Established	32/50 (64.0%)	8/19 (42.1%)
Cochin Hand Function Scale	7.0 _(0, 20)_, *n = 35*	0.0 _(0, 5)_, *n = 14*
mRSS	3.5 _(0, 16)_, *n = 50*	2.0 _(0, 7)_, *n = 19*
Disease characteristics		
Active digital ulcers	9/50 (18.0%)	2/19 (10.5%)
Previous digital ulcers	17/50 (34.0%)	4/19 (21.1%)
Joint synovitis	14/50 (28.0%)	4/19 (21.1%)
Clinical signs of arthritis on the hands	7/43 (16.3%)	1/15 (6.7%)
Joint contractures	25/43 (58.1%)	2/11 (18.2%)
Tendon friction rubs	5/49 (10.2%)	0/19 (0.0%)
Subcutaneous calcinosis of the hands	6/42 (14.3%)	0/15 (0.0%)

Variables are presented as mean ± SD for normal distribution or as median with the 1st and 3rd quartiles _(Q1, Q3)_ for non-normal distribution. Frequencies are shown as *x*/*y*: *x* = number of patients with item present, *y* = numbers of patients with accessible data; missing values can be calculated *n* − *y*

*mRSS* modified Rodnan Skin Score, *n* number, *SD* standard deviation

#### Low back pain is more prevalent in very early disease and not associated with disease-specific symptoms

Low back as main pain was more frequent in the group “very early” with 27.3% compared to 17.2% in “mild” and 12.3% in “established” (*p* = 0.196) (Table 2**,** Fig. 1). When not only major pain, but also overall pain was analysed, the proportion of patients reporting low back pain was significantly higher in the group “very early” (62.5% compared to 41.4% in “mild” and 29.2% in “established”; *p* = 0.010). As shown in Table 3, patients with low back pain showed less disease specific symptoms such as digital ulcers, joint contractures and arthritis in the hands than patients with hand pain. Accordingly, the median CHFS was significantly lower in patients with low back pain than in those without back pain. Regarding major pain, the median value for median pain intensity was 4 in patients with hand pain and 5 in patients with low back pain.

#### Low back pain is associated with advanced pain chronification

**Fig. 2:**
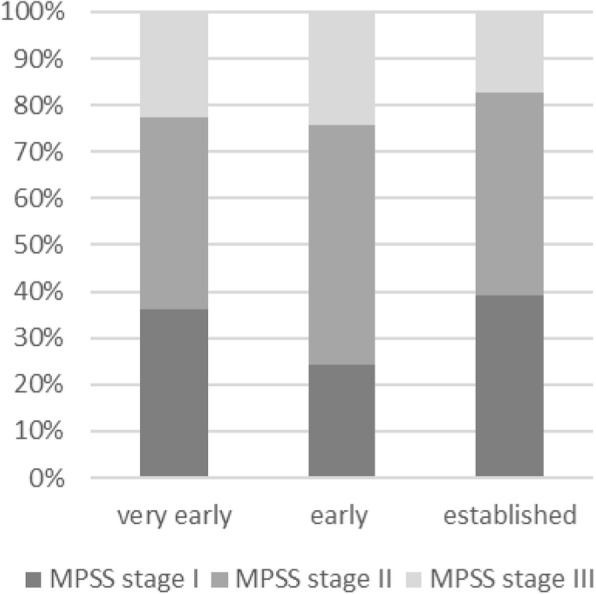
Distribution of pain chronification stages compared between the groups “very early”, “mild” and “established”, showing no association between pain chronification and disease severity

Regarding pain chronification, 40/115 patients (34.8%) were in stage I, 52/115 (45.2%) in stage II and 23/115 (20.0%) in stage III according to the MPSS. Contrary to our expectations, the proportion of patients with higher pain chronification stages did not increase with higher disease severity (Fig. 2). Instead, advanced pain chronification was more frequent in patients with low back pain. For patients with low back pain, the percentage of patients in MPSS stage III was higher with 42.1% compared to 18.0% in patients with hand pain. There were only 21.1% in MPSS stage I and 36.8% in stage II compared to 40.0% and 42.0%, respectively, in patients with hand pain. As major pain, only low back pain was significantly more frequent in higher chronification grades (*p* = 0.024); in stage III, more than a third of all patients reported low back pain as their major pain, and approximately 60% reported low back pain in general (Table 4). Regarding pain therapy, patients with low back pain showed—consistent with patients in advanced chronification stages—higher usage of analgesics, especially strong opioids, as well as antidepressants.

**Table 4:** Chronification stages

Pain chronification (MPSS), total of patients *n* = 115	Stage I, *n* = 40 (34.8%)	Stage II, *n* = 52 (45.2%)	Stage III, *n* = 23 (20.0%)
Age (years)	55.5 ± 15.7	58.6 ± 12.6	56.4 ± 13.4
Average pain intensity in the last 4 weeks (NRS)^1^	2.0 _(1, 3)_, *n = 39*	4.0 _(2, 5)_, *n = 50*	5.0 _(4, 7)_, *n = 23*
Most frequent localizations for main pain			
Back pain overall	9/40 (22.5%)	12/52 (23.1%)	9/23 (39.1%)
*Lumbar spine*	*4/40 (10.0%)*	*7/52 (13.5%)*	*8/23 (34.8%)*
Hand	20/40 (50.0%)	21/52 (40.4%)	9/23 (39.1%)
Most frequent localizations for overall pain			
Back	18/40 (45.0%)	31/52 (59.6%)	20/23 (87.0%)
*Lumbar spine*	*10/40 (25.0%)*	*20/52 (38.5%)*	*14/23 (60.9%)*
Hand	27/40 (67.5%)	45/52 (86.5%)	21/23 (91.3%)
Joint of the lower extremity	12/40 (30.0%)	35/52 (67.3%)	18/23 (78.3%)
Foot	9/40 (22.5%)	22/52 (42.3%)	9/23 (39.1%)

Variables are presented as mean ± SD for normal distribution or as median with the 1st and 3rd quartiles _(Q1, Q3)_ for non-normal distribution. Frequencies are shown as *x*/*y*: *x* = number of patients with item present, *y* = numbers of patients with accessible data; missing values can be calculated *n* − *y*

*MPSS* Mainz Pain Staging system, *n* number, *NRS* numeric rating scale, *SD* standard deviation

^1^Referring to major pain

#### Symptoms of anxiety and depression are more frequent in early stages of the disease and are associated with the presence of low back pain

Overall, 18 out of 107 (16.8%) patients with completed assessments showed a positive HADS score for anxiety, and 12 out of 106 (11.3%) patients had a positive HADS score for depression. As shown in Table 5, the “very early” group showed the highest percentage of positive scores for both with 25% each. In addition, the “very early” group showed more frequently pathological scores for well-being (30.0% compared to 10.3% in “mild” and 18.0% in “established”). As expected, higher chronification grades significantly correlated with higher amounts of positive HADS scores for anxiety (*p* < 0.001) as well as depression (*p* < 0.001) and with pathologically low scores for well-being as assessed by the MFHW (*p* = 0.004). From patients in MPSS stage III, 42.9% showed a positive HADS anxiety score and 28.6% a positive HADS depression score; almost 40% showed pathological scores for well-being (Table 6). Therefore, since also more of those patients showed advance chronification grades, patients with low back pain more often had positive HADS scores for anxiety (22.2%) and depression (16.7%) compared to patients with hand pain (8.9% and 6.7%, respectively), which is displayed in Table 7.

**Table 5:** Psychological factors according to disease manifestation

Subset of disease manifestation	Overall, *n* = 118	Established, *n* = 65 (55.1%)	Mild, *n* = 29 (24.6%)	Very early, *n* = 24 (20.3%)
HADS Anxiety Score				
Pathological	18/107 (16.8%)	10/61 (16.4%)	3/26 (11.5%)	5/20 (25.0%)
Borderline	21/107 (19.6%)	11/61 (18.0%)	6/26 (23.1%)	4/20 (20.0%)
HADS Depression Score				
Pathological	12/106 (11.3%)	7/60 (11.7%)	0/26 (0.0%)	6/20 (25.0%)
Borderline	13/106 (12.3%)	6/60 (10.0%)	4/26 (15.4%)	3/20 (15.0%)
MFHW ≤ 10	20/110 (18.2%)	11/61 (18.0%)	3/29 (10.3%)	6/20 (30.0%)

Variables are presented as mean ± SD for normal distribution or as median with the 1st and 3rd quartiles _(Q1, Q3)_ for non-normal distribution. Frequencies are shown as *x*/*y*: *x* = number of patients with item present, *y* = numbers of patients with accessible data; missing values can be calculated *n* − *y*

*HADS* Hospital Anxiety and Depression Scale, *MFHW* Marburger Fragebogen zum habituellen Wohlbefinden (Marburg questionnaire on habitual health findings), *n* number

**Table 6:** Psychological factors and therapy according to chronification stage

Pain chronification (MPSS), total of patients, *n* = 115	Stage I, *n* = 40 (34.8%)	Stage II, *n* = 52 (45.2%)	Stage III, *n* = 23 (20.0%)
HADS Anxiety Score			
Positive	1/37 (2.7%)	7/46 (15.2%)	9/21 (42.9%)
Borderline	6/37 (16.2%)	9/46 (19.6%)	6/21 (28.6%)
HADS Depression Score			
Positive	1/37 (2.7%)	4/45 (8.9%)	6/21 (28.6%)
Borderline	3/37 (8.1%)	4/45 (8.9%)	6/21 (28.6%)
MFHW ≤ 10	3/39 (7.7%)	8/48 (16.7%)	9/23 (39.1%)
Analgesic drug therapy overall	12/40 (30.0%)	30/52 (57.7%)	19/23 (82.6%)
*Analgesics WHO I (non-opioids)*	*12/40 (30.0%)*	*29/52 (55.8%)*	*16/23 (69.5%)*
*Analgesics WHO II (mild opioids)*	*1/40 (2.5%)*	*4/52 (7.6%)*	*7/23 (30.4%)*
*Analgesics WHO III (strong opioids)*	*0/40 (0.0%)*	*1/52 (1.9%)*	*5/23 (21.7%)*
Adjuvant pain medication			
Antidepressants	3/40 (7.5%)	9/52 (17.3%)	5/23 (21.7%)
Anticonvulsants	0/40 (0.0%)	2/52 (3.8%)	1/23 (4.3%)
Ambulant examination or therapy by			
Pain therapist	1/39 (2.6%)	1/50 (2.0%)	3/23 (13.0%)
Psychiatrist or psychologist	0/39 (0.0%)	3/50 (6.0%)	7/23 (30.4%)
Pain-related stationary treatment	4/34 (11.8%)	13/47 (27.7%)	13/20 (65.0%)
Pain-related stay in rehabilitation facility	2/33 (6.1%)	2/45 (4.4%)	7/19 (36.8%)

Variables are presented as mean ± SD for normal distribution or as median with the 1st and 3rd quartiles _(Q1, Q3)_ for non-normal distribution. Frequencies are shown as *x*/*y*: *x* = number of patients with item present, *y* = numbers of patients with accessible data; missing values can be calculated *n* − *y*

*HADS* hospital anxiety and depression scale, *MFHW* Marburger Fragebogen zum habituellen Wohlbefinden (Marburg questionnaire on habitual health findings), *MPSS* Mainz Pain Staging System, *n* number, *WHO* World Health Organization

**Table 7:** Psychological factors and therapy compared between hand and low back pain

Differences in main pain localization	Hand pain (*n* = 50)	Low back pain (*n* = 19)
HADS Anxiety Score		
Positive	4/45 (8.9%)	4/18 (22.2%)
Borderline	7/45 (15.6%)	3/18 (16.7%)
HADS Depression Score		
Positive	3/45 (6.7%)	3/18 (16.7%)
Borderline	4/45 (8.9%)	3/18 (16.7%)
MFHW ≤ 10	6/47 (12.8%)	6/19 (31.6%)
Analgesic drug therapy overall	22/50 (44.0%)	15/19 (78.9%)
*Analgesics WHO I (non-opioids)*	*21/50 (42.0%)*	*12/19 (63.2%)*
*Analgesics WHO II (mild opioids)*	*5/50 (10.0%)*	*4/19 (21.0%)*
*Analgesics WHO III (strong opioids)*	*2/50 (4.0%)*	*3/19 (15.8%)*
Adjuvant pain medication		
Antidepressants	4/50 (8.0%)	5/19 (26.4%)
Anticonvulsants	0/5 (0.0%)	1/19 (5.3%)

Variables are presented as mean ± SD for normal distribution or as median with the 1st and 3rd quartiles _(Q1, Q3)_ for non-normal distribution. Frequencies are shown as *x*/*y*: *x* = number of patients with item present, *y* = numbers of patients with accessible data; missing values can be calculated *n* − *y*

*HADS* Hospital Anxiety and Depression Scale, *MFHW* Marburger Fragebogen zum habituellen Wohlbefinden (Marburg questionnaire on habitual health findings), *n* number, *WHO* World Health Organization

## Discussion

Our study confirmed that pain is a central problem in SSc patients. The mean pain intensity was moderate, yet two thirds of the patients showed pain chronification. One in five patients met the highest stage of chronification. Higher grades of pain chronification were associated with higher HADS scores and higher usage of analgesics including strong opioids. The observation that patients with advanced pain chronification more frequently use medication, including opioids, has previously been shown during the validation of the MPSS [27]. Contrary to our expectations, pain intensity and chronification were not associated with increased disease severity. A surprisingly high number of patients reported low back pain as their major pain, especially in the early stages of the disease. Those patients also showed increased frequency for symptoms of anxiety and depression. Hand pain, being associated with typical scleroderma manifestations like ulcers, joint contractures and impaired hand function, gained importance with increasing disease severity.

In our study, 87% of the patients reported pain, which is very similar to a large descriptive study about pain in SSc that showed 83% of the patients were suffering from pain [6]. Two smaller studies found 75% [44] and 63% [7] of the patients reporting pain. It must be noted that in these studies, almost all patients included fulfilled the ACR 1980 criteria for SSc, consistent only with the “established” group in our study. None of the mentioned studies gathered information on pain localization or chronification. To our knowledge, no published study has yet examined the prevalence of back pain in SSc. Various studies examined depressive symptoms and their influencing factors in SSc patients [15, 16]. In our study, symptoms of anxiety and depression were significantly more frequent in patients with very early disease, which may be due to the increased frequency of low back pain in this group. Another possible explanation, amongst others, includes the recent diagnosis of SSc, which could also contribute symptoms of depression and anxiety, as previous studies have shown in cancer patients [45].

Regarding pain chronification, our results show similar [29, 46] or lower [27, 28, 47] extents of pain chronification compared to other studies that used the MPSS in pain cohorts. A recent study about ambulant and stationary pain therapy showed a very high extent of pain chronification, probably indicating an increasing number of patients with severe chronic pain in such therapy centres [48]. However, it must be taken into account that patients in those studies were collected from specialized pain clinics or practices, whilst only approximately 5% of the patients in our study reported seeing a pain specialist. Supporting our finding that main pain localized on the lower back was associated with more severe pain chronification, some of these studies showed that patients with back pain showed higher grades of pain chronification [28, 46].

Back pain is a widespread complaint in the general population; a large multiregional survey in Germany showed a 1-year prevalence of 75% [49], an investigation conducted by the Federal Republic of Germany showed that prevalence rates are increasing [50]. Whilst in the European guidelines for the treatment of acute non-specific low back pain it is stated that low back pain is usually self-limiting and about 90% the patients will recover within 6 weeks [51], other review studies found that approximately 60% of the patients still suffered from pain after 1 year [52, 53]. Low back pain leads to psychological problems for the individual due to its association with depression [54], but also to a heavy socioeconomic burden due to the high resulting costs [55, 56]. In order to avert these negative effects, early assessment of pain in all its dimensions and adequate multimodal pain treatment [57] are required. The effectiveness of multimodal pain therapy in patients with chronic back pain has been confirmed [58], showing positive effects for patients of all MPSS chronification stages. However, significantly less pain reduction was achieved in patients that have been suffering from back pain for more than 3 years, which underlines the importance of early pain assessment. These implications may also be applied to SSc patients with low back pain. Fibromyalgia frequently coexists with other rheumatologic diseases [59]. Low back pain was not specifically addressed in previous studies of SSc patients, yet one previous study found fibromyalgia—also representing a non-disease-specific symptom—to be considerably contributing to disability in SSc patient [60]. Therefore, the possibility of secondary fibromyalgia contributing to low back pain in SSc patients has to be taken into account.

A recent study about chronic pain in rheumatic diseases has shown a positive effect of multimodal pain therapy on the patient’s pain-related impairment as well as physical and mental well-being [61]. However, the study did not include patients with SSc, and the grade of pain chronification was not assessed.

Our study has several limitations. For some sub-analyses, there were missing data and the sample size was rather small. Due to the cross-sectional study design, no statements on long-term changes in the assessed symptoms can be made. Further, we did not record the source of hand pain; therefore, we could not differentiate between pain caused by ulcers, Raynaud’s phenomenon, arthritis or other symptoms. We also did not have additional information on the specific cause and associated morphological changes of the lower back pain in the patients, which needs to be addressed in further studies. In addition, we were not able to distinguish between nociceptive and neuropathic pain, since no validated screening instrument for neuropathic pain was used in the questionnaire. Furthermore, there was no data on the presence of fibromyalgia as a potential confounder for pain chronification available in our database. The fact that we included patients diagnosed with SSc that did not yet fulfil any classification criteria may be beneficial, since very early disease stages are considered in the study, though it makes our patient sample less comparable to previous publications, where usually the 1980 ACR classification criteria had to be fulfilled as an inclusion criterion. However, our study is of importance because it is—according to our knowledge—the first to conduct such an extensive pain analysis in patients with SSc, including frequency of pain localizations and the grading of pain chronification.

It remains unclear why low back pain is more frequent in the early stages of the disease in this study. A possible explanation might be the fact that with disease deterioration, other SSc-related pain symptoms such as hand pain gain priority. As selection bias cannot be ruled out, further studies examining the prevalence of back pain in SSc patients will be needed. However, our analysis has shown that back pain is a very severe problem in patients with SSc, as it is in the general population.

## Conclusions

Taken together, our study has important implications for the clinical care of SSc patients. To improve treatment and quality of life, early pain assessments should be conducted in order to detect or prevent pain chronification and associated psychological problems. Our study showed that low back pain is a major driver of pain in patients with very early disease. It is associated with higher pain chronification, depression and anxiety and has therefore major impact on well-being particularly in this patient group. It therefore needs particular attention in the care of patients with very early SSc.

## Data Availability

Some data for this study were extracted from the local EUSTAR database. Data are available upon reasonable request. All data concerning the results of the pain questionnaire are included in this article’s text and tables.

## Abbreviations

ACR: American College of Rheumatology
CHFS: Cochin Hand Function Scale
dSSc: Diffuse SSc
EUSTAR: European Scleroderma Trials and Research
HADS: Hospital Anxiety and Depression Scale
HRCT: High-resolution computed tomography
lcSSc: Limited cutaneous SSc
MFHW: Marburg questionnaire on habitual health findings
MPSS: Mainz Pain Staging System
mRSS: Modified Rodnan Skin Score
NRS: Numeric rating scale
SD: Standard deviation
SSc: Systemic sclerosis
WHO: World Health Organization
